# Magnetic exchange interaction between unpaired π- and d-electrons in nanographene-metal coordination complexes

**DOI:** 10.1093/nsr/nwaf033

**Published:** 2025-01-27

**Authors:** Deng-Yuan Li, Yuqiang Zheng, Ricardo Ortiz, Bing-Xin Wang, Yashi Jiang, Bingkai Yuan, Xin-Yu Zhang, Can Li, Liang Liu, Xiaoxue Liu, Dandan Guan, Yaoyi Li, Hao Zheng, Canhua Liu, Jinfeng Jia, Thomas Frederiksen, Pei-Nian Liu, Shiyong Wang

**Affiliations:** Key Laboratory of Natural Medicines, Department of Medicinal Chemistry, China Pharmaceutical University, Nanjing 211198, China; Key Laboratory of Artificial Structures and Quantum Control (Ministry of Education), TD Lee Institute, Shenyang National Laboratory for Materials Science, School of Physics and Astronomy, Shanghai Jiao Tong University, Shanghai 200240, China; Donostia International Physics Center (DIPC), San Sebastián 20018, Spain; Key Laboratory for Advanced Materials and Feringa Nobel Prize Scientist Joint Research Center, Frontiers Science Center for Materiobiology and Dynamic Chemistry, State Key Laboratory of Chemical Engineering, School of Chemistry and Molecular Engineering, East China University of Science & Technology, Shanghai 200237, China; Key Laboratory of Artificial Structures and Quantum Control (Ministry of Education), TD Lee Institute, Shenyang National Laboratory for Materials Science, School of Physics and Astronomy, Shanghai Jiao Tong University, Shanghai 200240, China; Suzhou Institute of Nano-Tech and Nano-Bionics, Chinese Academy of Sciences (CAS), Suzhou 215123, China; Key Laboratory for Advanced Materials and Feringa Nobel Prize Scientist Joint Research Center, Frontiers Science Center for Materiobiology and Dynamic Chemistry, State Key Laboratory of Chemical Engineering, School of Chemistry and Molecular Engineering, East China University of Science & Technology, Shanghai 200237, China; Key Laboratory of Artificial Structures and Quantum Control (Ministry of Education), TD Lee Institute, Shenyang National Laboratory for Materials Science, School of Physics and Astronomy, Shanghai Jiao Tong University, Shanghai 200240, China; Key Laboratory of Artificial Structures and Quantum Control (Ministry of Education), TD Lee Institute, Shenyang National Laboratory for Materials Science, School of Physics and Astronomy, Shanghai Jiao Tong University, Shanghai 200240, China; Hefei National Laboratory, Hefei 230088, China; Key Laboratory of Artificial Structures and Quantum Control (Ministry of Education), TD Lee Institute, Shenyang National Laboratory for Materials Science, School of Physics and Astronomy, Shanghai Jiao Tong University, Shanghai 200240, China; Hefei National Laboratory, Hefei 230088, China; Key Laboratory of Artificial Structures and Quantum Control (Ministry of Education), TD Lee Institute, Shenyang National Laboratory for Materials Science, School of Physics and Astronomy, Shanghai Jiao Tong University, Shanghai 200240, China; Hefei National Laboratory, Hefei 230088, China; Key Laboratory of Artificial Structures and Quantum Control (Ministry of Education), TD Lee Institute, Shenyang National Laboratory for Materials Science, School of Physics and Astronomy, Shanghai Jiao Tong University, Shanghai 200240, China; Hefei National Laboratory, Hefei 230088, China; Key Laboratory of Artificial Structures and Quantum Control (Ministry of Education), TD Lee Institute, Shenyang National Laboratory for Materials Science, School of Physics and Astronomy, Shanghai Jiao Tong University, Shanghai 200240, China; Hefei National Laboratory, Hefei 230088, China; Key Laboratory of Artificial Structures and Quantum Control (Ministry of Education), TD Lee Institute, Shenyang National Laboratory for Materials Science, School of Physics and Astronomy, Shanghai Jiao Tong University, Shanghai 200240, China; Hefei National Laboratory, Hefei 230088, China; Key Laboratory of Artificial Structures and Quantum Control (Ministry of Education), TD Lee Institute, Shenyang National Laboratory for Materials Science, School of Physics and Astronomy, Shanghai Jiao Tong University, Shanghai 200240, China; Hefei National Laboratory, Hefei 230088, China; Donostia International Physics Center (DIPC), San Sebastián 20018, Spain; Ikerbasque, Basque Foundation for Science, Bilbao 48013, Spain; Key Laboratory of Natural Medicines, Department of Medicinal Chemistry, China Pharmaceutical University, Nanjing 211198, China; Key Laboratory for Advanced Materials and Feringa Nobel Prize Scientist Joint Research Center, Frontiers Science Center for Materiobiology and Dynamic Chemistry, State Key Laboratory of Chemical Engineering, School of Chemistry and Molecular Engineering, East China University of Science & Technology, Shanghai 200237, China; Key Laboratory of Artificial Structures and Quantum Control (Ministry of Education), TD Lee Institute, Shenyang National Laboratory for Materials Science, School of Physics and Astronomy, Shanghai Jiao Tong University, Shanghai 200240, China; Hefei National Laboratory, Hefei 230088, China

**Keywords:** open-shell nanographene, magnetic exchange interaction, metal-organic coordination complexes, on-surface synthesis, scanning probe microscopy

## Abstract

The combination of open-shell nanographenes (NGs) and magnetic transition metals holds great promise for generating various new quantum phases applicable in spintronics and quantum information technologies. However, a crucial aspect in accomplishing this is to comprehend the magnetic exchange interactions between unpaired π- and d-electrons, a topic that has been seldom addressed. In this study, we focus on magnetic π-d exchange interactions between open-shell NGs and a magnetic coordination center of Fe or Co by employing scanning tunneling microscopy and spectroscopy. We synthesize two sets of NG-metal coordination complexes on a Au(111) substrate, secured by coordination bonds of carboxyl acid-Fe (Co). Through analysis of the excitation spectra, we observe a characteristic exchange coupling of 9 meV (5 meV) between the unpaired π-electron and the Fe (Co) d-shell electrons. Our experimental findings are qualitatively in agreement with multiconfigurational quantum chemistry calculations. This work provides evidence that a substantial magnetic exchange coupling can be achieved and engineered in metal-organic coordination systems, paving the way for designing and customizing extended radical metal-organic frameworks with precisely tailored magnetic properties.

## Teaser

This study demonstrates the achievement of a significant p-d magnetic exchange interaction in metal-nanographene coordination complexes, laying the groundwork for the design and customization of extended radical metal-organic frameworks with collective quantum magnetic properties.

## INTRODUCTION

The incorporation of magnetic metal centers into metal-organic frameworks (MOFs) provides an avenue for introducing magnetism into these materials [1–11]. However, in many MOFs, the magnetic exchange interaction between adjacent metal centers is exceedingly weak, with coupling strengths of a few μeV [12]. This weak magnetic exchange is primarily attributed to the significant separation between metal centers imposed by the organic ligands, resulting in distances much larger than typical atomic bond lengths. To overcome this limitation, the utilization of open-shell ligands presents a promising strategy. These open-shell ligands, with their unpaired π-electrons, can facilitate magnetic interactions with the magnetic metal centers, enabling the emergence of a wide range of phenomena, including topological phases, spin frustration, magnetic phase transitions, and potentially even room-temperature magnetism [13,14]. Despite their remarkable potential, the synthesis and characterization of radical MOFs pose challenges due to the inherent reactivity and instability associated with radicals.

Recently, a surface-assisted synthesis method has emerged as a powerful technique to directly create open-shell nanographene (NG) molecules on solid surfaces [15–17], opening up possibilities for fabricating radical MOFs. Open-shell NGs refer to nanoscale fragments of graphene with hydrogen-passivated edges hosting one or

multiple unpaired π-electrons [18]. These unpaired π-electrons are delocalized within the NG, giving rise to unique magnetic properties [19,20], including adjustable magnetic ground states [21], delocalized spin density distributions [19] and a prolonged spin-decoherence time [22]. By careful selection of precursor molecules and precise control of reaction conditions, a series of open-shell NGs and covalent oligomers have been successfully synthesized on solid surfaces [23–38]. To the best of our knowledge, the coordination of open-shell NGs with metal centers has not yet been achieved, although a π-d coupling has been observed in open-shell metallated porphyrin molecules [39]. Such coordination, combining unpaired electrons in their π-orbitals and metal centers with d-shell electronic configurations, holds the potential for an enhanced magnetic exchange interaction and collective quantum magnetism. Investigating the magnetic exchange interaction between the unpaired π- and d-electrons is thus essential for understanding and tailoring the properties of extended radical MOFs.

**Figure 1.**
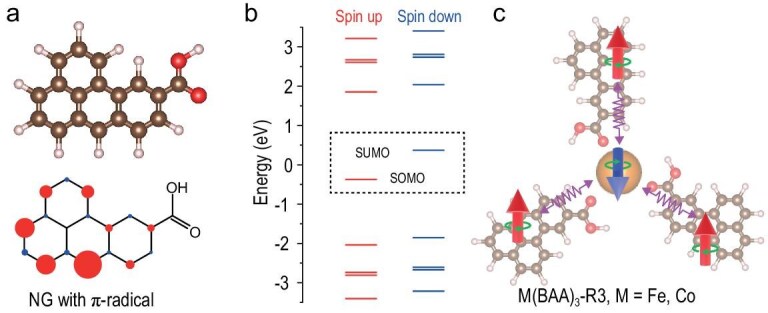
(a) Structure model (top) and DFT calculated π-electron spin density distribution (bottom) of the BAA radical derivative and (b) the corresponding DFT energy spectrum showing a singly occupied molecular orbital corresponding to an *S* = 1/2 ground state. Red: spin-up density; blue: spin-down density. (c) Structure model of an open-shell M(BAA)_3_-R3 coordination complex, where M = Fe, Co. Brown, red, off-white, and orange spheres in the structure models of Fig. 1a and c indicate the C, O, H, and metal atoms, respectively. The red upward and blue downward arrows in Fig. 1c represent the unpaired π- and d-electron spins, respectively.

In this study, we conduct a systematic investigation of the magnetic exchange interaction between an unpaired π-electron and the d-shell electrons of Fe and Co coordination centers using scanning tunneling microscopy and spectroscopy (STM/STS). Firstly, we fabricated a series of closed-shell NG-metal coordination complexes by co-depositing molecular precursors of 7a,11a-dihydro-7*H*-benzo[d, e]anthracene-10-carboxylic acid (BAA) and Fe (Co) atoms onto an Au(111) surface. This resulted in the formation of coordinated trimers and tetramers comprising three or four NG ligands, respectively, and one coordination metal atom. By utilizing STM tip-assisted atomic manipulation, we were able to selectively introduce the unpaired π-electron into the NG ligands by dissociating one hydrogen from the sp^3^ carbon. Our STS measurements revealed a magnetic coupling between the delocalized unpaired π-electron and the d-shell electrons of Fe (Co), with an exchange coupling strength of 9 (5) meV (Fig. 1c). To gain further insight, we performed multiconfigurational quantum chemistry calculations to elucidate the ground and excited states of these coordination complexes. These calculations demonstrate that the magnetic exchange interaction arises from the overlap between the non-bonding π-orbitals and the out-of-plane d*_yz_* and d*_xz_* orbitals of the metal center, with an excitation energy qualitatively consistent with our experimental observations. Our findings shed light on the magnetic exchange interaction between the unpaired π-electron and d-shell electrons, providing a foundation for designing and tailoring extended radical MOFs with desired magnetic properties. This knowledge is crucial for exploring their potential application in areas such as spintronics, information storage and quantum information [22,40,41].

## RESULTS AND DISCUSSION

Figure 1 illustrates the investigative strategy for studying the magnetic exchange interaction in NG-metal complexes. The selected NG possesses a spin ground state of *S* = 1/2, attributed to the sublattice imbalance of the graphene honeycomb lattice (Fig. 1a). This is confirmed by our spin-polarized density functional theory (DFT) calculations, which reveal a singly occupied molecular orbital and a delocalized spin density distribution, characteristic of an unpaired π-electron (Fig. 1a and b). To facilitate the exploration of the π-d magnetic exchange interaction, we intentionally introduce a carboxyl group at one side of the NG. This functional group can establish coordination bonds with magnetic metal atoms on surfaces, enabling the investigation of magnetic interactions (Fig. 1c).

**Figure 2.**
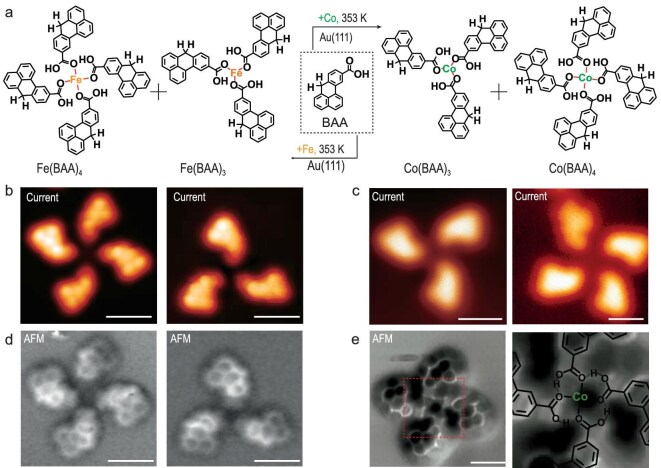
Formation and structural characterization of NG-Fe (Co) coordination complexes on Au(111). (a) Coordination reactions of precursor BAA and Fe or Co adatoms on Au(111). (b and c) Constant-height-current images of representative NG-Fe (Co) coordination complexes with rotational symmetry and homotactic configuration (b: Fe(BAA)_4_; Fe(BAA)_3_; c: Co(BAA)_3_; Co(BAA)_4_). (d) nc-AFM images (*f* = 24 K, amplitude: 30pm, bias: 2 mV) of Fe(BAA)_3_ and Fe(BAA)_4_. (e) nc-AFM image (*f* = 21 K, amplitude: 100 pm, bias: 10 mV) of Co(BAA)_4_ and the corresponding zoom-in nc-AFM image. Scale bars of all STM and constant-height-current images: 1 nm.

Initially, BAA was selected as a precursor for the *in situ* formation of metal-organic complexes on the Au(111) surface via a coordination bond between the carboxyl group and commonly found transition metal atoms such as Fe and Co (Fig. 2a). These complexes are anticipated to serve as excellent model systems for the creation of open-shell NG-metal coordination complexes on Au(111) through the manipulation of the methylene group using an STM tip. The advantageous compatibility between the growth of metal-organic complexes and the formation of the open-shell NG allows for the study of the magnetic exchange interaction between the unpaired π-electron and d-shell electrons on Au(111). We notice that BAA precursors can also coordinate with gold adatoms without detectable magnetic coupling (see Fig. S3).

**Synthesis of NG-Fe (Co) coordination complexes on Au(111).** The precursor BAA and magnetic transition metal atoms (Fe or Co) were deposited onto the Au(111) surface, followed by thermal annealing at 353 K for 5 min under ultra-high vacuum conditions. Subsequently, the samples were cooled down to ∼5 K for further characterization. Large-scale STM images revealed the presence of various aggregates, including clover-like (Fe(BAA)_3_ or Co(BAA)_3_), four-lobed (Fe(BAA)_4_ or Co(BAA)_4_), and other irregular structures adsorbed randomly on Au(111) (Fig. S1a and c). Statistical analysis showed that the combined proportion of clover-like and four-lobed aggregates in the Fe- or Co-containing Au(111) samples accounted for ∼60% and 80%, respectively, with four-lobed aggregates being more prevalent (Fig. S1b and d). Higher-resolution STM images (Fig. 2b and c) displayed two representative regular aggregates with homotactic configurations: one exhibiting 3-fold rotational symmetry and the other displaying 4-fold rotational symmetry. Notably, the metal centers of some coordination complexes are brighter, which can be attributed to the out-of-plane adsorption geometry of the center metal atom with enhanced mobility on surfaces.

Further insights were obtained through non-contact atomic force microscopy (nc-AFM) measurements [42], revealing that in Fe(BAA)_3_, Fe(BAA)_4_ and Co(BAA)_4_, the methylene group of BAA remained intact while the carboxylic acid group exhibited a planar adsorption conformation (Fig. 2d and e, right). These observed features combined with nc-AFM simulation (Fig. S2) suggested that the precursor BAA underwent a coordination reaction between the carboxyl group and the magnetic transition metal adatoms, leading to the formation of the desired NG-Fe (Co) coordination complexes (Fig. 2a).

**Figure 3.**
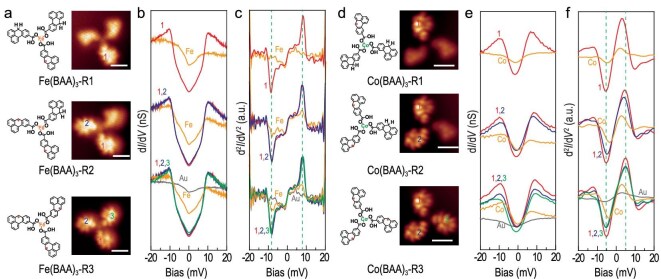
Electronic and magnetic characterization of representative open-shell NG-Fe (Co) coordination complexes with 3-fold rotational symmetry on Au(111). (a) Chemical structures and constant-height-current images. (b) d*I*/d*V* and (c) d^2^*I*/d*V* ^2^ spectra of open-shell NG-Fe coordination complexes Fe(BAA)_3_-R1, Fe(BAA)_3_-R2 and Fe(BAA)_3_-R3. (d) Chemical structures and constant-height-current images. (e) d*I*/d*V* and (f) d^2^*I*/d*V* ^2^ spectra of open-shell NG-Co coordination complexes Co(BAA)_3_-R1, Co(BAA)_3_-R2 and Co(BAA)_3_-R3. Scale bars: 1 nm.

**Magnetic exchange interaction of radical Fe(BAA)_3_ and Co(BAA)_3_ trimers.** To precisely generate unpaired π-electrons in the NG-Fe (Co) coordination complexes without altering their coordination structures, a stepwise STM tip-assisted atomic manipulation technique was employed [30,43]. This involved applying a voltage pulse of ∼3 V to homolyze one hydrogen atom from the methylene group in the organic ligands. Figure 3 showcases six representative open-shell NG-metal coordination complexes, comprising three open-shell NG-Fe coordination complexes (Fe(BAA)_3_-R_1_, Fe(BAA)_3_-R_2_, Fe(BAA)_3_-R_3_) and three open-shell NG-Co coordination complexes (Co(BAA)_3_-R_1_, Co(BAA)_3_-R_2_, Co(BAA)_3_-R_3_).

**Figure 4.**
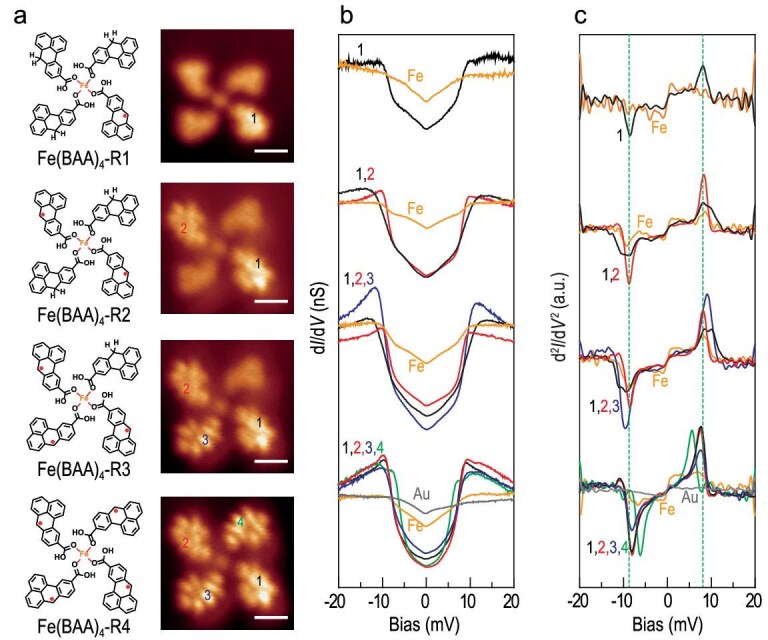
Electronic and magnetic characterization of representative open-shell NG-Fe coordination complexes with 4-fold rotational symmetry on Au(111). (a) Chemical structures and constant-height-current images. (b) d*I*/d*V* and (c) d^2^*I*/d*V* ^2^ spectra of open-shell NG-Fe coordination complexes Fe(BAA)_4_-R1, Fe(BAA)_4_-R2, Fe(BAA)_4_-R3 and Fe(BAA)_4_-R4. Scale bars of all constant-height-current images: 1 nm.

After dissociating one C–H bond from the sp^3^ carbon in one NG ligand by tip manipulation, an unpaired π-electron is introduced, producing a net spin of *S* = 1/2 in that NG, in agreement with Lieb's theorem [21]. As shown in Fig. 3a and d, the constant-height current images revealed that only the manipulated ligand exhibited distinct features due to the presence of a singly occupied molecular orbital hosting the generated unpaired π-electron in Fe(BAA)_3_-R_1_ and Co(BAA)_3_-R_1_. The low-energy d*I*/d*V* spectra taken at the open-shell NG ligands displayed two symmetric peaks near the Fermi level, which differed significantly from the Kondo peak observed in individual *S* = 1/2 magnetic NGs and Au(BAA)_3_R*_x_* (Fig. S3) [44]. These results suggest that the unpaired π-electron in Fe(BAA)_3_-R_1_ and Co(BAA)_3_-R_1_ is magnetically coupled with the d-electrons of the central metal atoms, with magnetic exchange energies around 9 and 5 meV, respectively. The difference in π-d magnetic exchange energies between Fe(BAA)_3_-R_1_ and Co(BAA)_3_-R_1_ may be attributed to the different number of unpaired d-electrons in Fe and Co (assuming the same oxidation state) that can couple and contribute to the magnetic exchange. In addition to the U-shape feature at 9 mV, a V-shaped dip is also observed in the Fe-coordinated complexes (Figs 3b and 4b), which is present at both the Fe site and nanographene sites. We attribute this dip feature to the magnetic anisotropy of Fe, similar to the anisotropy feature observed in Fe-metalated porphyrin [39].

Furthermore, Fe(BAA)_3_-R_2_ and Co(BAA)_3_-R_2_ with two open-shell NG ligands, each possessing an unpaired π-electron, were successfully generated and visualized using constant-height current imaging. The d*I*/d*V* and d^2^*I*/d*V* ^2^ spectra provided insights into the π-d magnetic exchange energies in the two different organic ligands. The exchange energies were found to be similar to those of Fe(BAA)_3_-R_1_ and Co(BAA)_3_-R_1_. Similarly, the constant-height current images of Fe(BAA)_3_-R_3_ and Co(BAA)_3_-R_3_ confirmed the formation of all open-shell NG ligands with π-d magnetic exchange energies consistent with those observed in Fe(BAA)_3_-R_1_ and Co(BAA)_3_-R_1_.

**Engineering magnetic exchange coupling strength.** Eight representative open-shell NG-Fe (Co) coordination complexes with 4-fold rotational symmetry and homotactic configuration were generated through stepwise STM tip manipulation and visualized using STM imaging and spectroscopy. As shown in Fig. 4a, the constant-height current images reveal the formation of open-shell NG-Fe coordination complexes with one, two, three and four unpaired π-electrons in Fe(BAA)_4_-R_1_, Fe(BAA)_4_-R_2_, Fe(BAA)_4_-R_3_ and Fe(BAA)_4_-R_4_, respectively. The corresponding d*I*/d*V* and d^2^*I*/d*V* ^2^ spectra (Fig. 4b and c) showed that all π-d magnetic exchange energies were close to those observed in radical Fe(BAA)_3_ trimers, indicating that the NG-Fe coordination interactions in coordinated trimers and tetramers are similar. As shown in Fig. S4, similar observations have been made for Co(BAA)_4_-R_1_, Co(BAA)_4_-R_2_, Co(BAA)_4_-R_3_ and Co(BAA)_4_-R_4_. The π-d magnetic coupling strength of tetramers is comparable to that in trimers (Fig. S4b and c). The irrelevance of the number of unpaired π-electrons regarding the exchange strength suggests that the magnetic coupling originates from the orbital overlap between the singly occupied π orbital and d-shell electrons, and hence interactions between π-radicals of different NGs are negligible.

**Figure 5.**
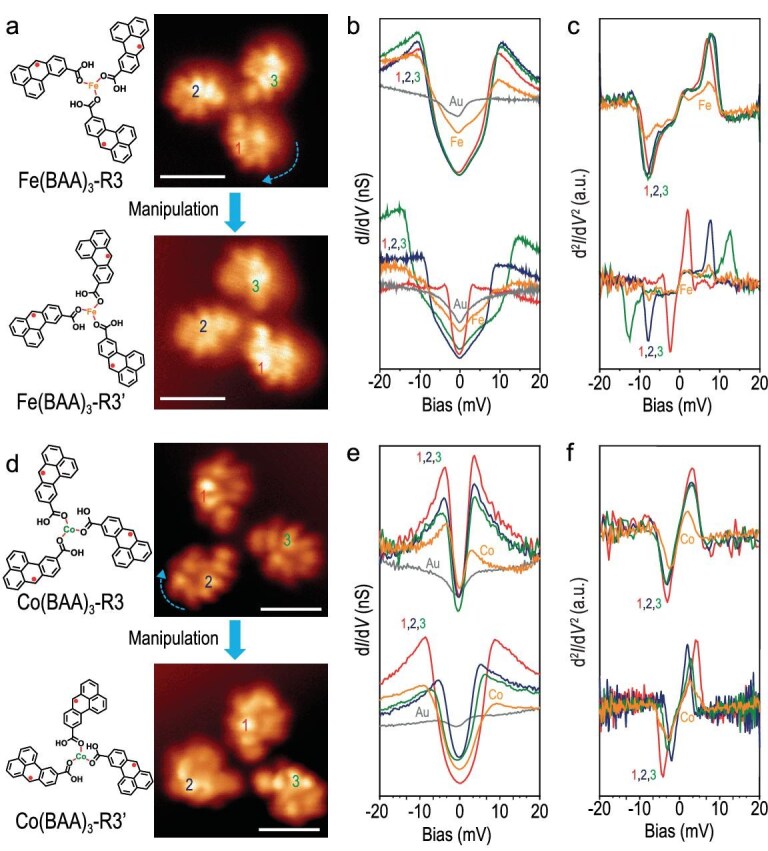
Engineering π-d exchange coupling strength. (a) Chemical structure and constant-height-current images of Fe(BAA)_3_-R3 before or after tip manipulation, and (b and c) the corresponding d*I*/d*V* and d^2^*I*/d*V* ^2^ spectra. (d) Chemical structure and constant-height-current images of Co(BAA)_3_-R3 before or after tip manipulation, and (e and f) the corresponding d*I*/d*V* and d^2^*I*/d*V* ^2^ spectra. Scale bars of all constant-height-current images: 1 nm.

The strength of the π-d exchange interaction is critically dependent on the π-d orbital overlap, which can be adjusted by modifying the π-d coordination bonds. To explore this, we conducted STM manipulation experiments to adjust the bond angle. As depicted in Fig. 5a–c, we performed tip manipulation experiments to transition Fe(BAA)_3_-R3 from a symmetric configuration to an asymmetric configuration. The results revealed that the symmetric Fe(BAA)_3_-R3, prior to tip manipulation, displayed almost the same spin-flip energy at 9 meV on the three different NGs. In contrast, the asymmetric Fe(BAA)_3_-R3 formed after tip manipulation exhibited three different spin-flip energies on the three different NGs, measuring at 15, 9 and 4 meV, respectively. These findings suggest that the coordination angle of the carboxylic acid group and the metal atom impacts the π-d orbital overlap, thereby altering the magnetic π-d exchange energy. Such measurements provide an effective means to engineer π-d exchange coupling strength in coordination complexes. We have conducted similar manipulation experiments on Co systems, and comparable observations have been obtained (Fig. 5d–f).

**Theoretical calculations of the magnetic exchange coupling between unpaired π- and d-electrons.** The multiconfigurational excitation spectra for the experimentally studied NG-metal complexes were calculated in the gas phase with the complete active space self-consistent field (CASSCF) method using the ORCA package [45]. First, the molecular geometry was relaxed with DFT, a def2-TZVP (triple-zeta valence polarization) basis, and the DFT-D3 method, to account for intramolecular van der Waals interactions. The NG-metal systems presented here have several bonds that are free to rotate and lead to different conformational isomers while keeping planarity (Figs S5 and S6). First, the C–COOH bond has two positions, and the isomer with the orientation of lower energy is different in the trimers with respect to the tetramers. Second, the rotation of the C–OH bond may orientate the hydrogen outwards or face the carbonyl oxygen of the adjacent ligand. It is the last scenario that was shown to be most stable in our calculations, suggesting the formation of intramolecular H-bonds as a stabilizing factor of the whole molecule, with rather short donor–acceptor distances (≈2.46, 1.58 and 2.38 Å for Fe(BAA)_3_-R_3_, Fe(BAA)_4_-R_4_ and Co(BAA)_3_-R_3_, respectively) [46].

**Figure 6.**
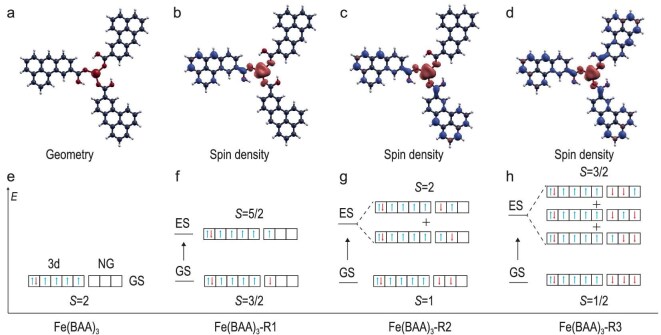
Theoretical analysis for free-standing 3-fold NG-Fe coordination complexes. (a) Structure model of Fe(BAA)_3_. (b–d) Structure models of Fe(BAA)_3_-R1, Fe(BAA)_3_-R2 and Fe(BAA)_3_-R3 superimposed with the ground-state spin density (colors blue and red stand for the sign) and (e–h) the corresponding electronic configuration of the ground and first-excited states.

With the relaxed geometry, an additional DFT calculation was done with a def2-SVP basis (BP86), where the presence of unpaired electrons triggered the emergence of magnetism at the NG ligands (Fig. 6a–d). The orbitals of the latter calculation were employed to start the multiconfigurational CASSCF methodology. First, the self-consistent field process is converged with a minimal Complete Active Space (CAS) that includes natural orbitals with a fractional occupation that correspond to the non-bonding orbitals from the NGs, and the 4s and 3d orbitals of the central metal atom. The number of electrons was chosen for a charge of +2 of the whole system, since other options resulted in the double occupation of one or more NG non-bonding orbitals, or formed empty orbitals, which, contrary to the experiment, would remove the spectroscopic signal in that part of the system. Because of technical limitations regarding the self-consistent convergence of bigger Active Spaces, next we converged the CAS just with one self-consistent field iteration, as in the complete active space configuration interaction method, including additional orbitals and electrons until their fractional occupation was above 1.6 and below 0.08 electrons.

Our results show that for each depassivated NG, an additional natural orbital with occupation close to 1 converged in the CAS, corresponding to the unpaired π-electrons with a strong localization at the NGs, as well as an additional spin-flip excited state following the ground state. Since the studied systems are either *C*_3_ or *C*_4_ symmetric, these excited states consist in the combination of the spin-flips of individual unpaired π-electrons (Fig. 6e–h). Hence the first excited state would be seen as an inelastic step in the d*I*/d*V* curve at the same bias for every monoradical NG, in agreement with the experimental observations. The main source of magnetic exchange is the coupling between unpaired π-electrons and unpaired d-electrons. Because Co has one less unpaired d-electron than Fe, assuming the same oxidation number, the resulting magnetic exchange can be anticipated to be smaller.

In our calculations, the first excitation state in all the studied systems was found to be within the range of 1–12 meV, in agreement with experimental results. As illustrated in Fig. 6, our chosen CAS set includes the 3d orbitals of Fe and the singly occupied orbitals of NG. This selection of orbitals effectively captures the nature of π-d magnetic coupling. We have summarized the CASSCF wave function of the NG non-bonding orbitals, highlighting the contribution of each d orbital. Our findings indicate that in the CASSCF wave function, the natural orbitals corresponding to the unpaired electrons in the NGs predominantly couple with the out-of-plane d*_yz_* and d*_xz_* orbitals (Table S1). Additionally, we calculated the ground and excited states of Co complexes, finding a slightly weaker π-d exchange coupling strength, consistent with experimental observations (Fig. S7).

## CONCLUSION

We synthesized two sets of open-shell NG-Fe (Co) coordination complexes on Au(111) by combining the coordination chemistry of monoradical carbon platforms functionalized with a carboxylic acid bound to Fe (Co) atoms and atomic manipulation techniques. Through STM/STS measurements, we investigated the magnetic exchange interaction between an open-shell NG and a magnetic Fe (Co) coordination center. The excitation spectra revealed a magnetic exchange coupling of 9 meV (5 meV) between the unpaired π-electrons and the Fe (Co) d-electrons, which were found to be in good agreement with our multiconfigurational quantum chemistry calculations. These findings highlight the significant magnetic exchange interaction achievable in metal-organic coordination systems, presenting opportunities for further exploration of topological properties and quantum magnetism in 2D MOFs that possess both π- and d-shell electrons.

## ADDITIONAL NOTES

During the preparation of this manuscript, we become aware of a recent work that also reports the STM/STS study on a similar system [47].

## Supplementary Material

nwaf033_Supplemental_File
