# Supplementary material for: Magnetic exchange interaction between unpaired π- and d-electrons in nanographene-metal coordination complexes
Source: Natl Sci Rev. 2025 Jan 27;12(4):nwaf033. doi: 10.1093/nsr/nwaf033 (PMC11887856; doi:10.1093/nsr/nwaf033)
Supplement: nwaf033_Supplemental_File [file nwaf033_supplemental_file.pdf]

Supplementary Information for

Magnetic Exchange Interaction Between Unpaired π- and d- Electrons in Nanographene-Metal Coordination Complexes

Deng-Yuan Li[1,#,*], Yuqiang Zheng[3,#], Ricardo Ortiz[4,#,*], Bing-Xin Wang[2], Yashi Jiang[3], Bingkai Yuan[5], Xin-Yu Zhang[2], Can Li[3], Liang Liu[3,6], Xiaoxue Liu[3,6], Dandan Guan[3,6], Yaoyi Li[3,6], Hao Zheng[3,6], Canhua Liu[3,6], Jinfeng Jia[3,6], Thomas Frederiksen[4,7], Pei-Nian Liu[1,2,*], Shiyong Wang[3,6,*]

[1]Key Laboratory of Natural Medicines, Department of Medicinal Chemistry, China Pharmaceutical University, Nanjing, 211198, P. R. China
[2]Key Laboratory for Advanced Materials and Feringa Nobel Prize Scientist Joint Research Center, Frontiers Science Center for Materiobiology and Dynamic Chemistry, State Key Laboratory of Chemical Engineering, School of Chemistry and Molecular Engineering, East China University of Science & Technology, Shanghai, 200237, P. R. China
[3]Key Laboratory of Artificial Structures and Quantum Control (Ministry of Education), TD Lee Institute, Shenyang National Laboratory for Materials Science, School of Physics and Astronomy, Shanghai Jiao Tong University, 800 Dongchuan Road, Shanghai 200240, P. R. China
[4]Donostia International Physics Center (DIPC) – UPV/EHU, 20018 San Sebastián, Spain
[5]Suzhou Institute of Nano-Tech and Nano-Bionics, Chinese Academy of Sciences (CAS), Suzhou 215123, P. R. China
[6]Hefei National Laboratory, Hefei 230088, P. R. China
[7]Ikerbasque, Basque Foundation for Science, 48013 Bilbao, Spain

[#]These authors contributed equally.

*Corresponding Authors: dengyuanli@cpu.edu.cn, roc6493@gmail.com, liupn@cpu.edu.cn, shiyong.wang@sjtu.edu.cn.

The supplementary information includes:

1. Synthesis of precursor S3

2. General procedure for the experimental measurements and theoretical calculations S6

3. Supplementary Figures S8

1. Synthesis of precursor

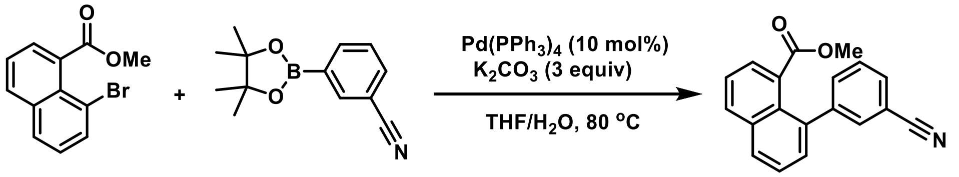

S1 S2 S3

A solution of methyl 8-bromo-1-naphthoate (S1, 158 mg, 0.6 mmol), 3-(4,4,5,5-tetramethyl-1,3,2-dioxaborolan-2-yl)benzonitrile (S2, 165 mg, 0.72 mmol), $Pd(PPh_3)_4$ (69 mg, 0.06 mmol), K_2CO_3 (249 mg, 1.8 mmol) in THF (4 mL) and H_2O (2 mL) was added in a 25 mL sealed tube under argon atmosphere. The mixture was stirred at 40 ℃ for 12 h. The resulting mixture was cooled to room temperature and extracted with DCM. The organic phase was separated and washed with H_2O, and dried over anhydrous Na_2SO_4. The purification was performed by a preparation plate with silica gel (silica, PE:EA = 25:1) to afford the product methyl 8-(3-cyanophenyl)-1-naphthoate (S3, 120 mg, 84%). ^{1}H NMR (400 MHz, $CDCl_3$, 25 ^{o}C): δ 7.97 (dd, J_1 =8.24 Hz, J_2 =1.00 Hz, 1H), 7.88 (d, J =8.16 Hz, 1H), 7.65-7.68 (m, 2H), 7.53-7.59 (m, 3H), 7,45-7.49 (m, 2H), 7.38 (dd, J_1 =7.12 Hz, J_2 =1.12 Hz, 1H), 3.16 (s, 3H); ^{13}C NMR (150 MHz, $CDCl_3$, 25 ^{o}C): δ 169.76, 144.13, 137.18, 134.84, 133.8, 132.21, 132.17, 130.80, 130.71, 130.57, 129.50, 129.32, 129.29, 127.86, 126.10, 125.19, 118.75, 112.57, 51.88; HRMS (EI, TOF): calcd for $C_{19}H_{13}NO_2^+$ $[M]^+$: 287.0946, found: 287.0944.

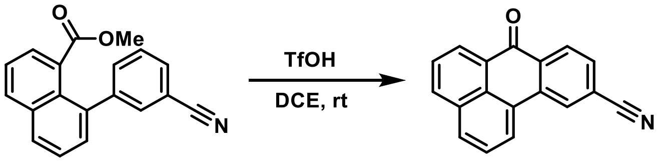

S3 S4

A solution of methyl 8-(3-cyanophenyl)-1-naphthoate (S3, 158 mg, 0.55 mmol) in DCM (6 mL) were added into a 25 mL sealed tube, and trifluoromethanesulfonic acid (0.44 mL, 4.95 mmol) was then added dropwise under nitrogen atmosphere. After the resulting mixture was stirred overnight at room temperature, the reaction was quenched with a saturated solution of $NaHCO_3$ and extracted with DCM. The organic phase was washed with water and dried over Na_2SO_4. The purification was performed by a preparation plate with silica gel (silica, PE:EA = 10:1) to afford the product 7-oxo-7*H*-benzo[de]anthracene-10-carbonitrile (S4, 35 mg, 25%). ^{1}H NMR (400 MHz, $CDCl_3$, 25 ^{o}C): δ

8.73 (dd, J_1 = 7.36 Hz, J_2 = 1.24 Hz, 1H), 8.59 (d, J = 1.24 Hz, 1H), 8.53 (d, J = 8.16 Hz, 1H), 8.40 (d, J = 7.36 Hz, 1H), 8.24 (dd, J_1 = 8.04 Hz, J_2 = 0.96 Hz, 1H), 8.05 (d, J = 8.12 Hz, 1H), 7.76-7.80 (m, 1H), 7.68-7.73 (m, 2H); ^{13}C NMR (150 MHz, $CDCl_3$, 25 ^{o}C): δ 182.64, 136.82, 136.10, 133.48, 133.18, 131.75, 130.74, 130.64, 129.09, 128.21, 128.01, 127.83, 127.23, 126.89, 125.11, 124.96, 118.45, 116.85; HRMS (EI, TOF): calcd for $C_{18}H_9NO^+$ $[M]^+$: 255.0684, found: 255.0682.

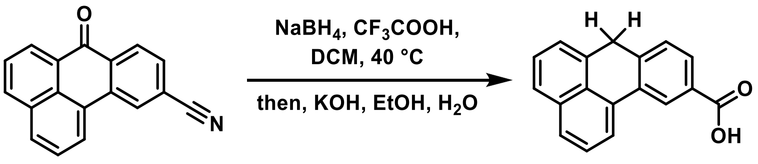

S4 BAA

Sodium borohydride (29.5 mg, 0.78 mmol), trifluoroacetic acid (2 mL) were added into a 25 mL sealed tube. After the mixture was stirred at room temperature for 20 min, a solution of 7-oxo-7*H*-benzo[*de*]anthracene-10-carbonitrile (40 mg, 0.156 mmol) in DCM (10 mL) was added. The resulting mixture was heated to 40 °C for 12 h. The reaction was cooled to room temperature and quenched with a saturated solution of $NaHCO_3$ and extracted with DCM. The organic phase was washed with H_2O (3×20 mL), and dried over anhydrous Na_2SO_4. The purification was performed by a preparation plate with silica gel (silica, PE) to afford the product 7*H*-benzo[*de*]anthracene-10-carbonitrile (13 mg, 35%). Then, a solution of 7*H*-benzo[*de*]anthracene-10-carbonitrile (36 mg, 0.15 mmol) and KOH (336.6 mg, 6 mmol) in EtOH (2 mL) and H_2O (2 mL) was added in a 25 mL sealed tube under N_2. The reaction mixture was heated and refluxed. After 12 h, the reaction was cooled to room temperature and extracted with EA. The organic phase was washed with H_2O and dried over anhydrous Na_2SO_4. The purification was performed by a preparation plate with silica gel (silica, EA) to afford the product 7*H*-benzo[*de*]anthracene-10-carboxylic acid (BAA, 12 mg, 35%). ^{1}H NMR (600 MHz, DMSO-d_6, 25 ^{o}C): δ 13.04 (br, COOH), 8.64 (s, 1H), 8.15 (d, J = 7.32 Hz, 1H), 7.87 (dd, J_1 = 7.92 Hz, J_2 = 1.56 Hz, 1H), 7.84 (d, J = 8.16 Hz, 1H), 7.75 (d, J = 8.16 Hz, 1H), 7.56 (t, J = 7.74 Hz, 1H), 7.50 (t, J = 7.26 Hz, 1H), 7.44-7.48 (m, 2H), 4.64 (s, 2H); ^{13}C NMR (150 MHz, DMSO-d_6, 25 ^{o}C): δ 167.75, 139.65, 134.01, 132.76, 132.44, 130.09, 130.04, 129.93, 128.97, 128.61, 128.48, 127.00, 126.81, 125.97, 125.29, 124.69, 119.67, 34.15; HRMS (EI, TOF): calcd for $C_{18}H_{12}O_2^+$ $[M]^+$: 260.0837, found: 260.0833.

^{1}H NMR (600 MHz, $CDCl_3$) of 7*H*-benzo[*de*]anthracene-10-carboxylic acid (BAA)

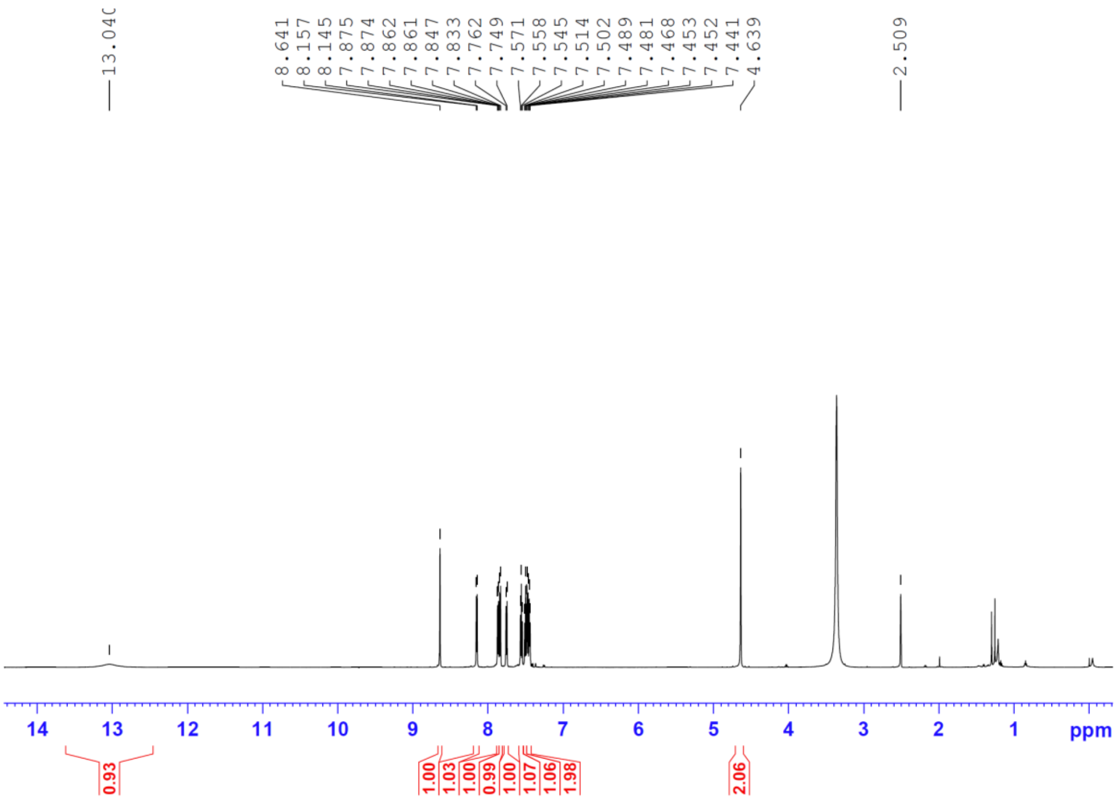

^{13}C NMR (150 MHz, $CDCl_3$) of 7*H*-benzo[*de*]anthracene-10-carboxylic acid (BAA)

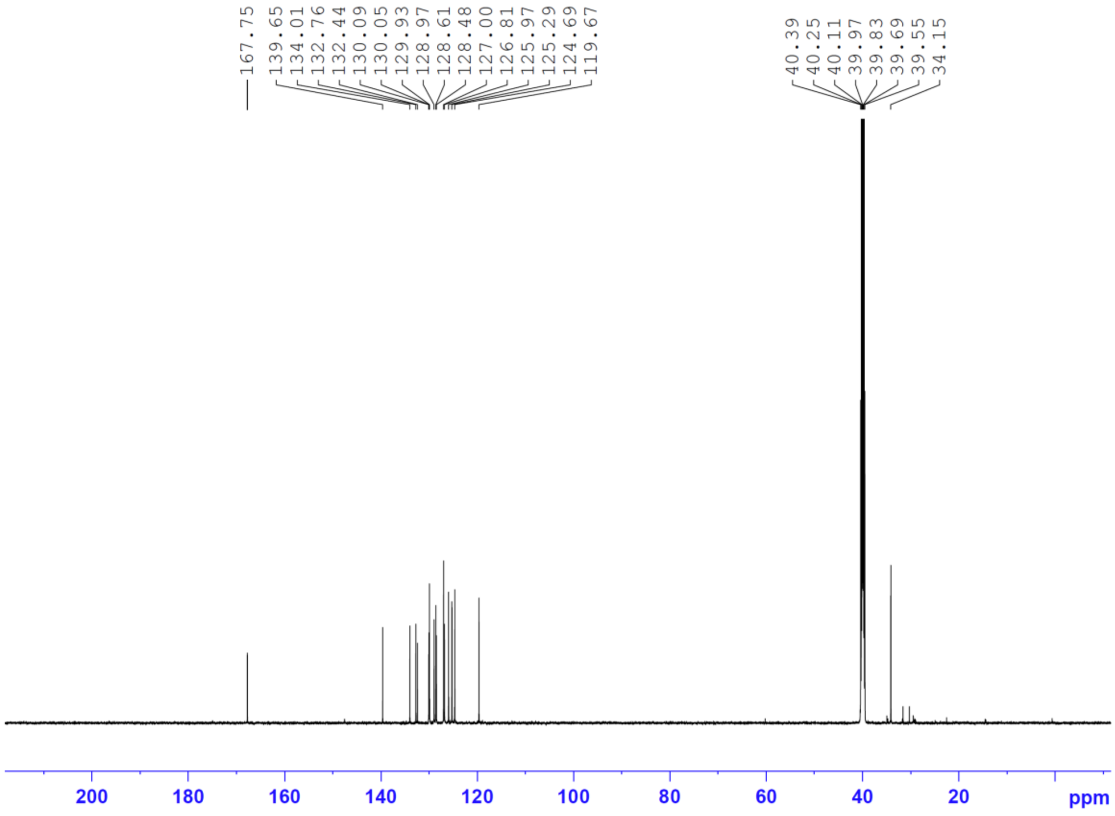

2. General procedure for the experimental measurements and theoretical calculations

Sample preparation and STM/nc-AFM measurement. A commercially available low-temperature Unisoku Joule-Thomson scanning probe microscope (1.2 K) operated at ultrahigh vacuum(3×10^{-10} mbr) was used for all sample preparation and characterization. The Au(111) single crystals were cleaned using repeated cycles of Ar^+ sputtering and subsequent annealing to 950 K to obtain atomically flat terraces. The cleanliness of the crystals was checked by scanning with the STM before molecular deposition. Precursor BAA and Fe (Co) were thermally co-deposited on the clean Au(111) surface at room temperature and annealed to 80 ℃ for 5 minutes. Precursor BAA was evaporated on the surface from a quartz crucible and the sublimation temperature was approximately 120 ^{o}C. The Fe (Co) was deposited on the surface using an e-beam evaporator. Then, the sample was transferred to a cryogenic scanner at 1.2 K for characterization. Carbon monoxide molecules were dosed onto the cold sample around 9 K (1×10^{-8} mbar, 1 minute). To achieve ultra-high spatial resolution, CO molecule was picked up from the Au(111) surface to the apex of a tungsten tip. A quartz tuning fork with a resonant frequency of 26 kHz has been used in nc-AFM measurements. A lock-in amplifier (531 Hz, 0.1-1 mV modulation) has been used to obtain dI/dV spectra. The STM, nc-AFM images, and STS measurements were taken at 1.2 K, and the data were processed with the WSxM software.

Theoretical calculations.

The theoretical calculations were carried out with the ORCA package. First, a geometric relaxation was performed with DFT, using the BP86 density functional, the def2-TZVP basis and the RI-J approximation for the Coulomb integrals. The DFT-D3 method was also considered in order to include the intramolecular H-bonds. In order to keep the C_3 symmetry in the trimers we added the invariance of the O-M-O angles as a constraint, as well as the planarity of the molecule.

A relaxation was done for several conformational isomers, among which the most stable was always that with a maximum number of H-bonds (3 and 4 for the trimer and tetramer, respectively). In addition to this, there is another isomer regarding the rotation of the C-COOH bond, and whose relative stability depends on the number of ligands.

Next, with the relaxed geometry we did an additional DFT calculation with the def2-SVP basis, obtaining non-negligible spin density everytime an sp^3 carbon was depassivated and more stable solutions with antiferromagnetic coupling. The resulting orbitals were used to initialize the CASSCF methodology with a minimum CAS that included the non-bonding orbitals of the monoradical nanographenes, and the d-shell orbitals and 4s orbital of the metal. Then, the number of electrons was chosen to be that of the 2+ oxidation state of the metal, since it was the only oxidation state where the monoradical nanographenes held one electron only in the non-bonding orbitals. This is CAS(9,9), CAS(10,10), CAS(10,9) and CAS(11,10) for $Fe(BAA)_3$-R3, $Fe(BAA)_4$-R4, $Co(BAA)_3$-R3 and $Co(BAA)_4$-R4, respectively.

3. Supplementary Figures

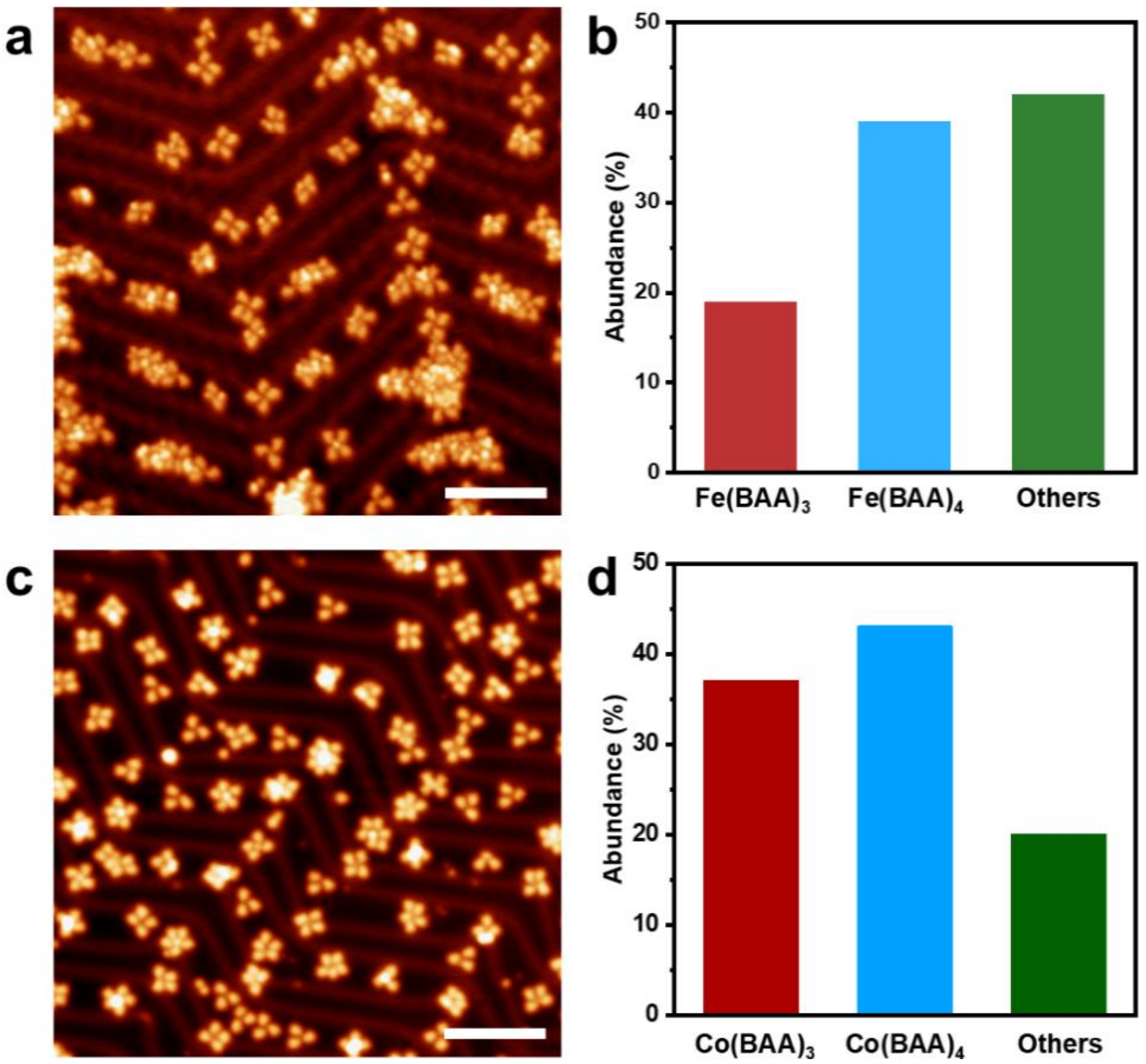

Supplementary Figure 1. (a and c) Large-scale STM image of (a) nanographene-Fe or (c) -Co coordination complexes on Au (111) after annealing at approximately 353 K and (b and d) the corresponding statistical analysis. Scale bars: (a and c) 10 nm. Scanning parameters: (a) U = -1 V, I = 10 pA. (c) U = 1 V, I = 10 pA.

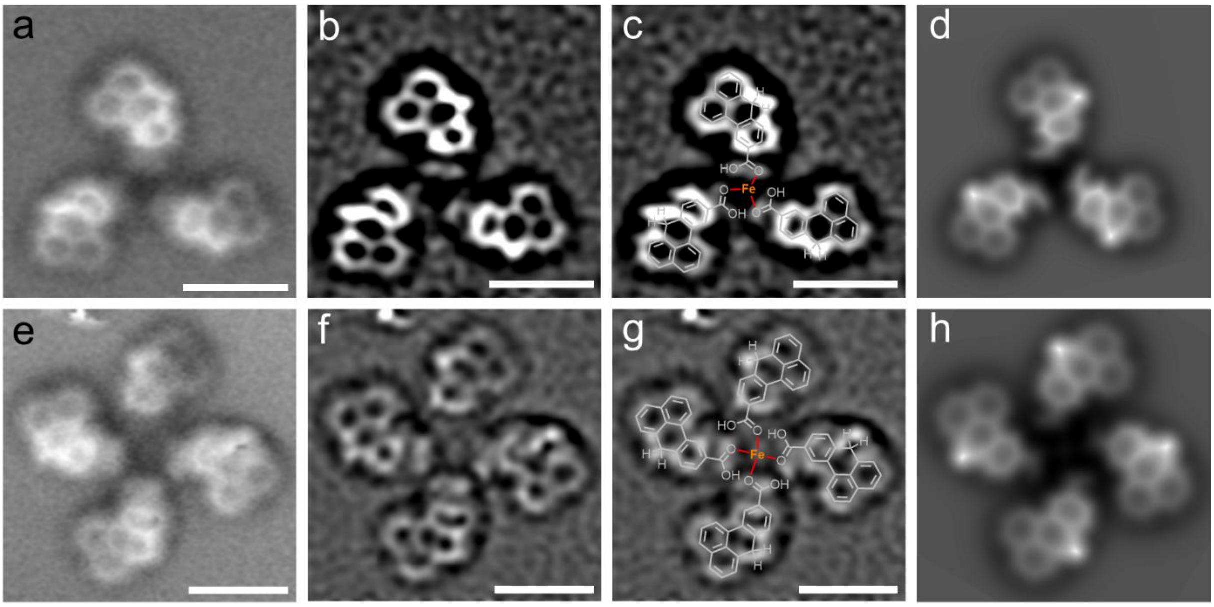

Supplementary Figure 2. (a and e) nc-AFM images of $Fe(BAA)_3$ and $Fe(BAA)_4$ with rotational symmetry, the (b, c, f, and g) Laplace-filtered nc-AFM images without and with chemical structure, and (d and h) the corresponding nc-AFM simulations. Scale bars: 1 nm. Tip heights: (a) z = −261 pm with respect to STM setpoint of 0.5 V, 10 pA on Au(111). (e) z = −278 pm with respect to STM setpoint of 0.2 V, 10 pA on Au(111).

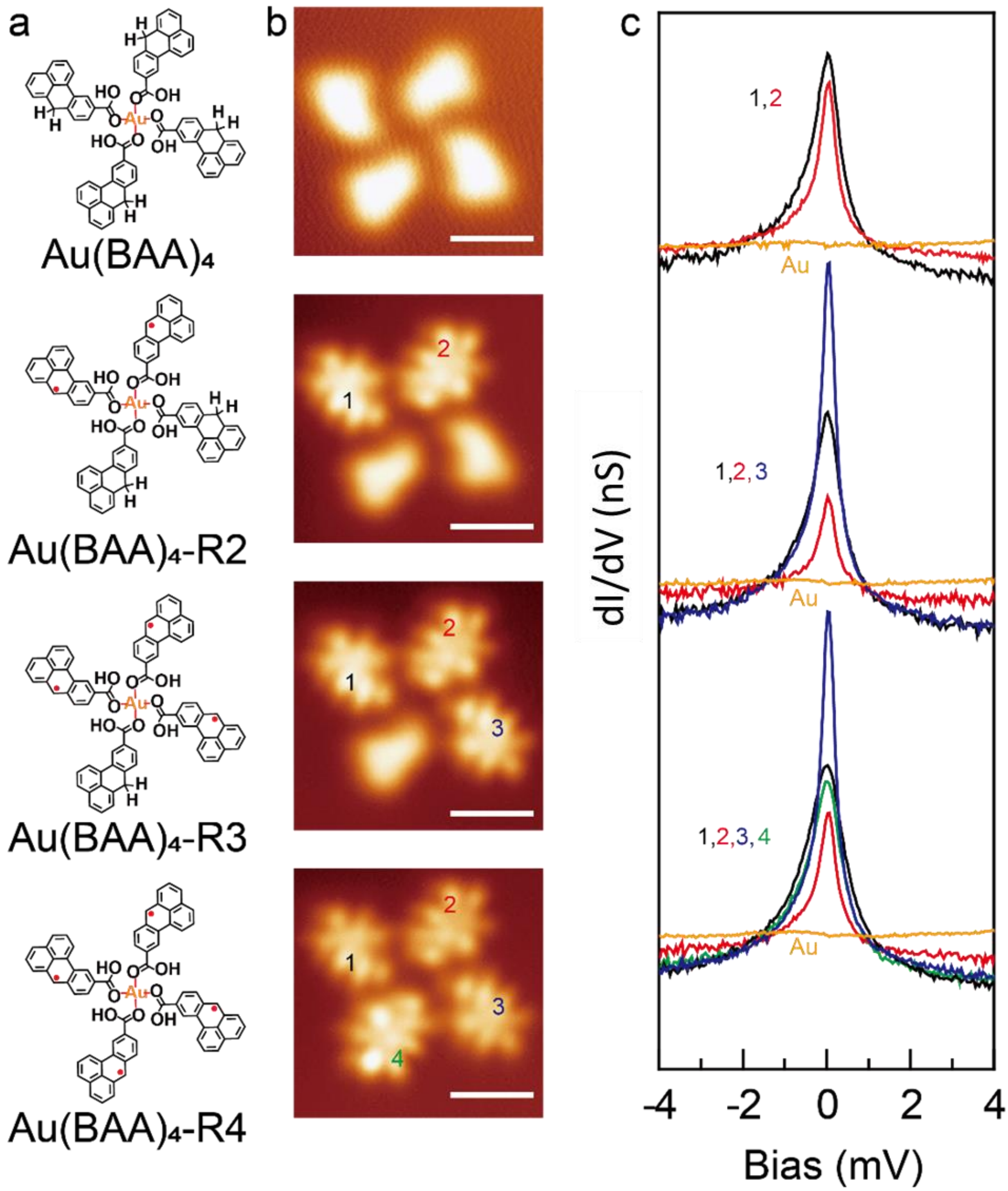

Supplementary Figure 3. (a) Chemical structures, (b) constant-height current images, and (c) dI/dV spectra of nanographene-Au coordination complexes Au(BAA)$_4$, Au(BAA)$_4$-R2, Au(BAA)$_4$-R3, and Au(BAA)$_4$-R4. Scale bars of all constant-height current images: 1 nm.

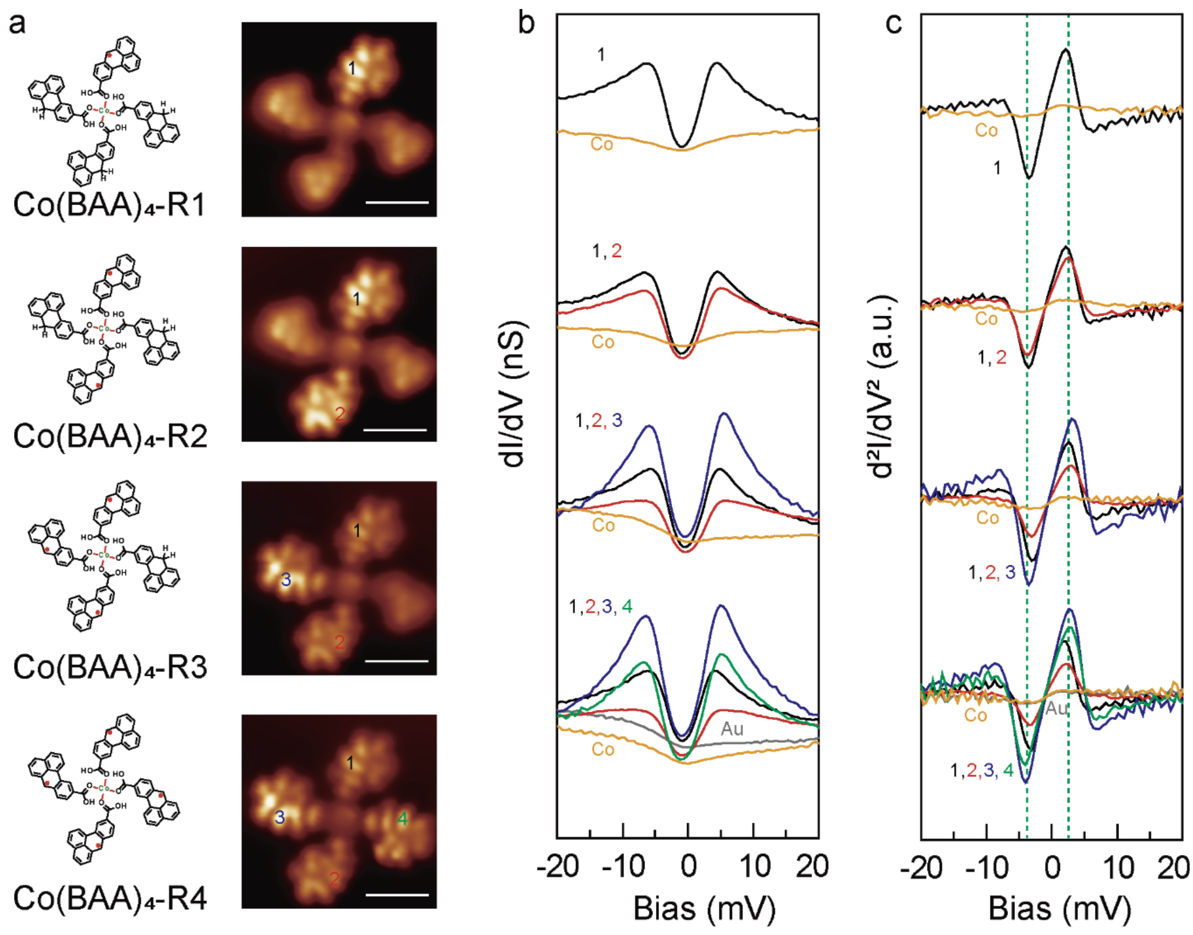

Supplementary Figure 4. Electronic and magnetic characterization of representative open-shell NG-Co coordination complexes with four-fold rotational symmetry on Au(111). (a) Chemical structures, constant-height current images, (b) dI/dV and (c) dI^2/dV^2 spectra of open-shell NG-Co coordination complexes $Co(BAA)_4$-R1, $Co(BAA)_4$-R2, $Co(BAA)_4$-R3, and $Co(BAA)_4$-R4. Scale bars of all constant-height current images: 1 nm.

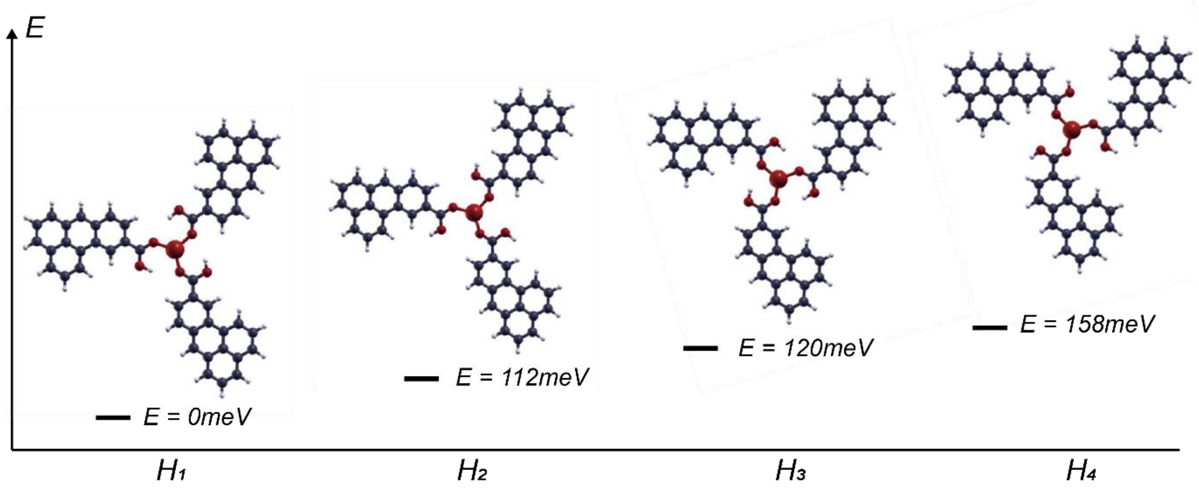

Supplementary Figure 5. DFT-calculated relative energies for $Fe(BAA)_3$ with different intramolecular hydrogen bonds.

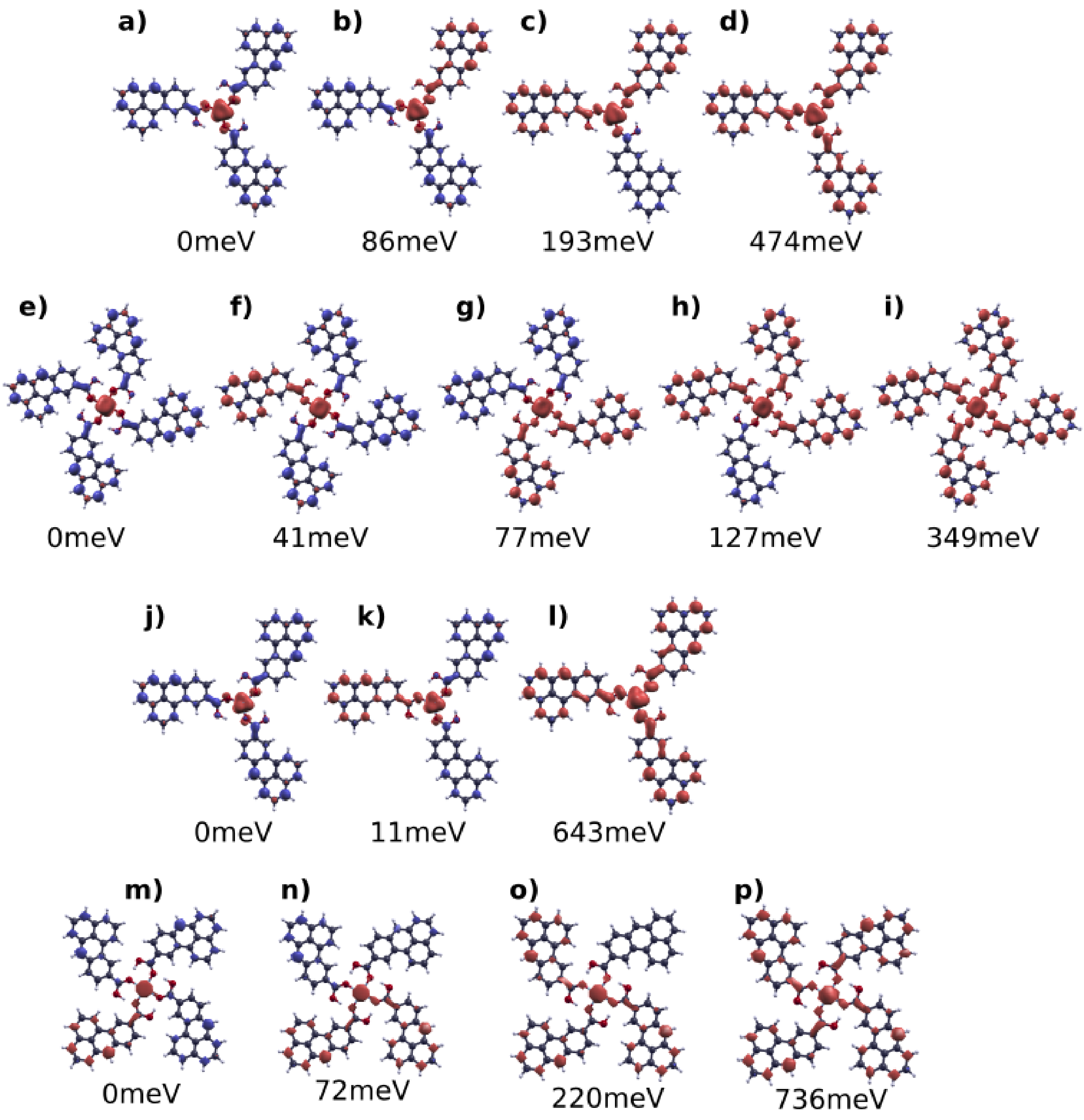

Supplementary Figure 6. Spin states and density distribution calculated with DFT for (a-d) $Fe(BAA)_3$-R3, (e-i) $Fe(BAA)_4$-R4, (j-l) $Co(BAA)_3$-R3 and (m-p) $Co(BAA)_4$-R4. Colors stand for the sign.

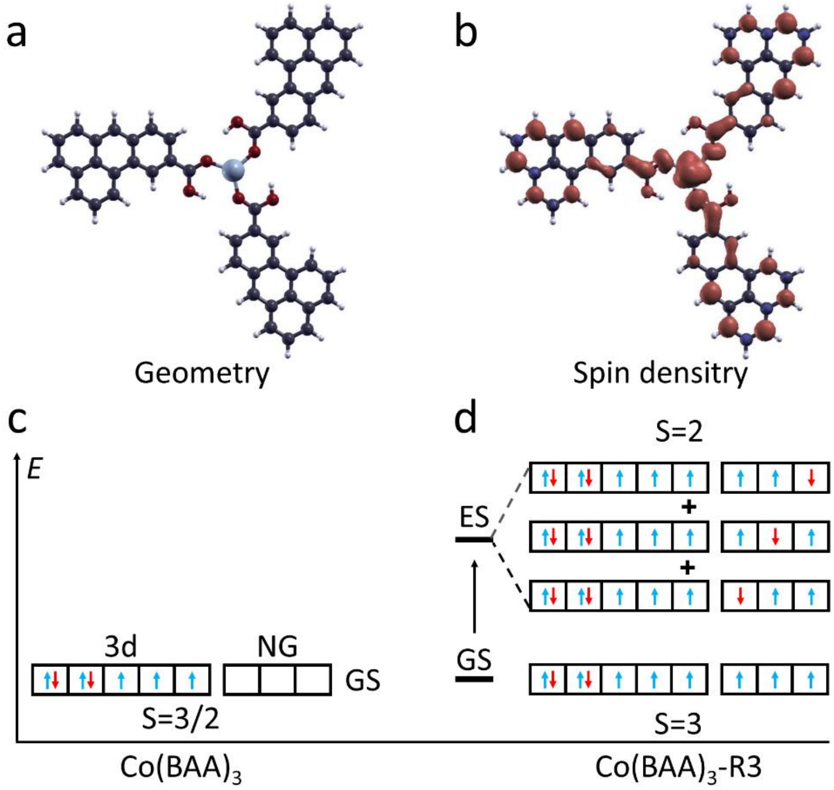

Supplementary Figure 7. Theoretical analysis for free-standing three-fold NG-Co coordination complexes. (a) Structure model of Co(BAA)$_3$. (b) Structure models of Co(BAA)$_3$-R3 superimposed with the ground-state spin-density (colors blue and red stand for the sign) and (c and d) the corresponding electronic configuration of the ground and first-excited states.

Supplementary Table 1. The CASSCF wave function of the nanographene non-bonding orbitals with the contribution of each d orbital.

non-bonding orbital	dyz	dxz	dz^2	dx^{2}y^2	dxy
Ob1	51%	23%	6%	9%	10%
Ob2	61%	34%	3%	0.4%	1%
Ob3	25%	66%	4%	2%	4%
